# Update on the Pathogenic Mechanisms of Isocyanate-induced Asthma

**DOI:** 10.1097/wox.0b013e3181625d8c

**Published:** 2008-01-15

**Authors:** Gyu-Young Hur, Sung-Jin Choi, Seung-Youp Shin, Sang-Ha Kim, Hae-Sim Park

**Affiliations:** 1 Department of Allergy and Rheumatology, Ajou University School of Medicine, YoungtongGu, WonchonDong, San-5, Suwon, Korea; 2 Department of Otolaryngology, College of Medicine, KyungHee University, Seoul; 3 Department of Internal Medicine, Yonsei University Wonju College of Medicine, Wonju, Korea

**Keywords:** asthma, genetic polymorphism, HLA, specific IgE, specific IgG, toluene diisocyanate

## Abstract

**Background**

The pathogenic mechanisms of toluene diisocyanate (TDI)-induced asthma are not understood clearly.

**Methods**

In this review, we summarized recent data to further understand the pathogenic mechanisms through recent approach of molecular genetic studies, and specific antibody responses to cellular mechanisms.

**Results**

Genetic polymorphism studies suggested the involvement of oxidant/antioxidant-related genes and neurogenic inflammatory response genes. A high-resolution technique of HLA allele suggested some HLA genetic markers presenting the phenotype of TDI-induced asthma. The new volatile type of TDI-human serum albumin conjugate could improve specific immunoglobulin E and immunoglobulin G bindings in enzyme-linked immunosorbent assay studies for identifying TDI-induced asthma patients. The role of bronchial epithelial cells and cytokeratins derived from epithelial cells could be involved in autoantibody productions, suggesting that an autoimmune mechanism may be involved in airway inflammation. Neutrophil activation and oxidant/antioxidant-related mechanisms were also suggested.

**Conclusion**

The pathogenic mechanism of TDI-induced asthma is complicated as various humoral and cellular mechanisms are combined and involved differently on an individual basis. Therefore, additional efforts for further elucidation should follow.

## 

Although more than 300 causative agents of asthma have been reported, isocyanate, especially toluene diisocyanate (TDI), is the most prevalent cause of occupational asthma worldwide. Although incidences are varied depending on the forms and types of isocyanate, it is generally reported that 5% of TDI-exposed workers could develop TDI-induced asthma [1]. The annual incidence rate of isocyanate-induced asthma was 1.8% in TDI production facilities [2]. The follow-up study on TDI-induced asthma demonstrated that 50% of TDI-induced asthmatic patients had experienced persistent asthmatic symptoms even after cessation of exposure to isocyanate [3]. The pathogenic mechanism of occupational asthma is complicated by the fact that both immunologic and non-immunologic pathways may be involved, depending upon the causative agent [3-6]. In addition, several environmental factors, including the nature of the causative agent and the level and mode of exposure, affect the pathogenesis of occupational asthma. In cases involving low-molecular-weight chemicals, particularly TDI, both immunoglobulin E (IgE)- and non-IgE-mediated pathways have been implicated [3-7]. Several studies examining the role of specific IgG antibodies against isocyanate have yielded controversial results [3-5]. This review summarizes our current understanding of the pathogenic mechanisms of TDI-induced asthma and outlines a series of questions that must be addressed to further our understanding of the pathogenesis of isocyanate-induced asthma.

## Genetic mechanisms of isocyanate-induced asthma

### HLA allele studies

A European study of 142 patients with TDI-induced asthma and 50 asymptomatic exposed controls demonstrated that HLA class I alleles were not significantly associated with TDI-induced asthma [8]. However, a comparison of TDI-induced asthma subjects with asymptomatic exposed controls using high-resolution techniques demonstrated that 1 HLA class II allele, DQB1*0503, and 1 haplotype, DQB1*0201-0301, were significantly associated with TDI-induced asthma [9,10]. These results were later refuted by data from a German population, showing no association between the disease and the HLA class II allele [11]. In a Korean population, we used a high-resolution sequencing method to compare a number of HLA class I and II alleles in 55 TDI-induced asthma patients with those in 47 asymptomatic exposed subjects and 95 unexposed healthy nonatopic controls; the HLA haplotype DRB1*15-DPB1*05 was found to be a susceptibility marker for the development of TDI-induced asthma among exposed workers [12]. To resolve the differences between our results and those of the other groups mentioned previously, additional studies using a larger cohort of TDI-induced asthma patients in different ethnic groups are needed.

### Genetic polymorphism studies

The genes for glutathione S-transferase [13] and N-acetyltransferase [14] are believed to confer susceptibility to, or protection against, TDI-associated asthma. Bernstein et al [15] suggested a gene-to-environment interaction with *IL4RA, CD14*, and *IL13*. Based on studies showing the involvement of neurogenic inflammation in TDI-induced asthma, we used a single base extension to screen for 2 single-nucleotide polymorphism of neurokinin 2 receptor (NK2R) gene, 7853C > T and 11424G > A, in 70 patients with TDI-induced occupational asthma, 59 asymptomatic exposed controls, and 93 unexposed healthy controls [16]. No significant differences were noted in the allele, genotype, or haplotype frequencies of the 2 single-nucleotide polymorphism among the 3 groups. However, those TDI-exposed workers with the NK2R 7853CC genotype had higher serum levels of vascular endothelial growth factor than did those with the CT or TT genotype. We speculate that the NK2R 7853CC genotype may contribute to an increase in the serum level of vascular endothelial growth factor, resulting in airway inflammation after exposure to TDI. Additional studies are needed to investigate other candidate genes and gene-to-environment interactions in people of various ethnicities.

### Role of specific IgE antibodies against isocyanate in occupational asthma

Several investigators have detected IgE antibodies specific for TDI-human serum albumin (HSA) conjugate in the sera of workers showing a positive bronchial challenge response to TDI, with a reported prevalence of 0% to 50% of workers [3,4,7]. Maestrelli et al [17] demonstrated that the bronchial mucosa of TDI-induced asthma patients contained increased numbers of cells expressing interleukin 5 (IL-5) and IL-4. Similarly, we found that 13% of TDI-induced asthma patients had specific IgE antibodies, [18] although subsequent enzyme-linked immunosorbent assay (ELISA) inhibition tests using 3 different TDI-HSA conjugates prepared under the same conditions revealed different inhibition patterns [19]. We recently demonstrated that the sensitivity of the specific IgE antibodies could be increased to 44% when a volatile type of TDI-HSA conjugate was used instead of a conventional liquid conjugate, [20] indicating that antigen quality affects the ability to detect specific antibodies. As shown in Table 1, the prevalence of specific IgE antibodies varied according to the antigen used and the population studied. These findings suggest that IgE bindings to the antigenic determinant of the TDI-HAS conjugate can differ from 1 individual to another; thus, if we improve the TDI-protein conjugate further, we should be able to identify additional specific antibodies. This may explain the variable results obtained in prevalence studies of serum-specific IgE antibodies against TDI-HSA conjugate in patients with TDI-induced asthma.

**Table 1:** Determination of Specific IgE and IgG Antibodies in Subjects With Isocyanate-induced Asthma

		Specific IgE (%)		Specific IgG (%)	
		
Authors (yr)	Assay	Se	Sp	Se	Sp
Butcher BT et al (1980) [42]	RAST	19	--	--	--
Pezzini A et al (1984) [43]	RAST	39	--	--	--
Keskinen H et al (1988) [44]	RAST	20	--	--	--
Cartier A et al (1989) [21]	ELISA	31	97	72	76
Karol MH et al (1994) [45]	RAST	3	93	3	93
Tee RD et al (1998) [7]	RAST ratio > 2	28	92	--	--
	RAST ratio > 3	20	100	--	--
Park HS et al (1999) [18]	ELISA	14	92	46	92
Bernstein DI et al (2002) [28]	ELISA	21	89	47	74
Ye YM et al (2006) [20]	ELISA	43.9	--	30.3	--

RAST indicates radioallergosorbent test; ELISA, enzyme-linked immunosorbent assay; Se, sensitivity; Sp, specificity.

Thus, it is certain that IgE-mediated responses contribute to the development of asthmatic symptoms in TDI-induced asthmatic patients; however, additional studies are needed to develop a more effective TDI-HSA conjugate and to investigate the role of non-IgE-mediated mechanisms.

### Pathogenic role of specific IgG antibodies

A few studies have suggested that specific IgG antibodies against hexamethylene diisocyanate (HDI)-HSA and methylene diphenyl diisocyanate (MDI)-HSA conjugates play a pathogenic role in occupational asthma based on an association between IgG antibodies and the results of bronchoprovocation tests (BPTs), [21] whereas other investigators have suggested that specific IgG antibodies against MDI are merely indicators of MDI exposure [22]. We also reported the prevalence of serum-specific IgG antibodies against TDI-HSA conjugates [18]. The serum level of specific IgG antibodies was significantly higher in subjects showing a positive response in a TDI-BPT (46%) compared with subjects exhibiting a negative TDI-BPT response (7.7%), subjects with allergic asthma (0%), and unexposed healthy controls (0%). The prevalence of specific IgG did not depend significantly upon the type of asthmatic response, the presence of specific IgE antibodies against TDI-HSA conjugate, or atopic status. Given these data, the presence of IgG antibodies against TDI-HSA conjugate may indicate exposure to TDI and may be associated with a patient's TDI-BPT results.

### The cellular immune response

Inflammatory reactions involving eosinophils, mast cells, and T lymphocytes, especially those bearing IL-2 receptors, [17,23] and increased cytokine production by T_h_2 cells occur in the bronchial mucosae of patients with TDI-induced asthma, as in patients with allergic asthma. Other immune responses to diisocyanate-related asthma include lymphoproliferative responses and cytokine/chemokine production with exposure to isocyanate [24,25]. Immunohistochemical finding comparing the bronchial mucosa between patients with TDI-induced asthma and those with allergic asthma has revealed significantly higher numbers of mast cells and neutrophils in patients with TDI-induced asthma but no significant difference in the number of T cells [26]. A significant correlation was also found between the numbers of neutrophils and mast cells. With regard to T-cell cytokine secretion, interferon-γ production was observed in both peripheral mononuclear cells and T-cell lines collected from subjects newly diagnosed as having TDI-induced asthma [24]. A few studies have demonstrated the role of CD8^+ ^T lymphocytes in the airway mucosa of patients with TDI-induced asthma, and a specific T-cell line was successfully derived from T cells in the airway mucosa of a patient with TDI-induced asthma [27]. In addition, it was shown that isocyanate-induced monocyte chemoattractant protein-1 (MCP-1) production by peripheral blood mononuclear cells may be used to differentiate between isocyanate-induced asthma patients and asymptomatic exposed controls [25,28]. These data suggest that T_h_2 lymphocytes and other inflammatory cells, such as eosinophils, mast cells, and activated neutrophils, may contribute to the development of TDI-induced bronchoconstriction. A role of CD8^+ ^T lymphocytes was suggested.

### Epithelial cells and autoimmune mechanisms

Mounting evidence suggests that diisocyanates are able to bind airway epithelial cell proteins, resulting in airway inflammation with cytokine and chemokine production and cellular recruitment [29]. The toxicity of diisocyanates toward airway epithelial tissues has also been reported, and MDI and HDI prepolymers were shown to induce airway epithelial barrier dysfunction that was partly associated with altered glutamine levels [30,31]. The effect of TDI on the expression of lung cytokine P450 enzymes has also been documented [32]. We attempted to culture human bronchial epithelial cells, Beas-2B, with TDI-HSA conjugates to identify the source of the IL-8 detected in the sputum of TDI-induced asthma patients [33]. The production of IL-8 and regulated upon activation in normal T cell expressed, and secreted (RANTES) was significantly increased in a dose-dependent manner after exposure to a peripheral mononuclear cell culture supernatant derived from a TDI-induced asthma patient, and this effect was suggested to be mediated by epidermal growth factor receptor and p38 mitogen-activated protein [34].

Recent microarray analyses of epithelioid cells demonstrated increased expression of cytokeratin (CK) 19 in cultured bronchial epithelial cells after TDI exposure. The prevalence of serum IgGs against CK-19 was significantly higher in TDI-induced asthma patients than in asymptomatic exposed controls or normal controls, [35] and the degree of airway hyperresponsiveness to methacholine was more severe in patients with serum CK-specific IgG [36]. The CKs are normally located in the intracellular space, but may be able to access the immune system upon epithelial damage or cell death. The precise mechanism of their production is unknown, although several studies have suggested that CKs are solubilized by proteolysis during apoptosis and can subsequently enter the circulation, where they may stimulate the formation of new antibodies. These findings indicate that among patients with TDI-induced asthma, those who are most susceptible to epithelial damage by TDI exposure may develop CK-specific IgGs.

### Neutrophil activation and oxidant/antioxidant mechanisms

The bronchoalveolar lavage fluid of isocyanate-induced asthma patients, especially those with latent asthmatic responses, showed neutrophilia [37]. The number of neutrophils was significantly higher in the bronchial mucosa of TDI-induced asthma patients than in allergic asthma patients in our study [26]. The myeloperoxidase level, a neutrophil activation marker, was increased in the airway secretions of TDI-induced asthma patients after TDI challenge, and this increase was accompanied by an increase in IL-8 and leukotriene B4 production [38,39]. This suggests that the release of IL-8 and leukotriene B4 after exposure to TDI contributes to neutrophilic infiltration into the airway mucosa of patients with TDI-induced asthma.

A remarkable increase in exhaled nitric oxide was detected after exposure to isocyanate in TDI-induced asthma patients [40]. Reduced levels of antioxidants were detected in the bronchial epithelial cells of patients exposed to TDI [41]. Considering the critical role of neutrophils in oxidant-induced injury in the asthmatic airway, oxidant/antioxidant-induced inflammation caused by activated neutrophils combined with reduced antioxidant content in the airway mucosa may contribute to the pathogenesis of TDI-induced asthma.

## Perspectives

Recent studies demonstrate that the pathogenic mechanism of TDI-induced asthma is complicated as various humoral and cellular mechanisms are combined and involved differently on an individual basis. More efforts should be devoted to increase the sensitivity of specific antibody detection techniques with a better TDI-HSA conjugate, and to study the cellular and molecular genetic mechanisms.

Notes

Supported by a grant from the Korean Health 21 R&D Project, Ministry of Health and Welfare, R.O.K. (A 050571).
